# Determination of 2-Pentanol Enantiomers via Chiral GC-MS and Its Sensory Evaluation in Baijiu

**DOI:** 10.3390/foods11172584

**Published:** 2022-08-26

**Authors:** Lisha Hu, Shuyi Qiu, Yifeng Dai, Luqin Tian, Chaoyang Wei

**Affiliations:** 1 Guizhou Province Key Laboratory of Fermentation Engineering and Biopharmacy, School of Liquor and Food Engineering, Guizhou University, Guiyang 550025, China; 2 Beijing Advanced Innovation Center for Food Nutrition and Human Health, Beijing Technology and Business University, Beijing 100048, China

**Keywords:** Baijiu, 2-pentanol, chirality, enantiomer, sensory analysis

## Abstract

The enantiomeric contents of 2-pentanol of Baijiu were analyzed by liquid-liquid extraction (LLE) coupled with gas chromatography-mass spectrometry (GC-MS) using β-cyclodextrin as a chiral stationary phase. In this study, the average enantiomeric ratios *R:S* were 72:28, 64:36, and 94:6 in soy sauce aroma-type Baijiu (SSB), strong aroma-type Baijiu (STB), and light aroma-type Baijiu (LTB), respectively, and only *(R)*- configuration was found in rice aroma-type Baijiu (RTB). The highest enantiomeric concentration of 2-pentanol was found in STB. *(R)*-2-pentanol dominated in 48 Baijiu studied, and the concentration of *(R)*-2-pentanol was higher than that of the *(S)*-configuration. The results showed that the enantiomers of 2-pentanol were discrepant in different aroma types of Baijiu, and it may be the result of differences in raw materials, environment, and production processes. The 2-pentanol enantiomers had different odor characteristics, with different olfactory thresholds in pure water and 46% ethanol solutions by sensory analysis. *(R)*-2-pentanol was described as paint, rubber, grease, while the *(S)*-form had mint, plastic, and pungent notes. The olfactory thresholds of *(R)*- and *(S)*-form were 163.30 mg/L and 78.58 mg/L in 46% ethanol and 12.62 mg/L and 3.03 mg/L in pure water, respectively. The different enantiomeric distribution and aroma characteristics of the 2-pentanol enantiomers in Baijiu could be a potential marker for determining adulteration.

## 1. Introduction

Chiral molecules are molecules that are not overlapped with their mirror images, and a chiral compound and its mirror image are known as enantiomers [1]. Since the late 1970s, the determination of enantiomeric concentrations and ratios in foods by chiral analysis has been crucial in food safety, quality control, authenticity, geographical origin, and sensory properties [2,3]. For example, *(S)*-ethyl lactate has been reported to be a marker of microbial contamination in wine [4], and the *R/S* enantiomer ratio of less than 90/10 in 1,2-propanediol in wine could determine wine adulteration [5]. The enantiomeric ratio of 1,2-propanediol in Baijiu could be used as a potential marker to distinguish the aroma types of Baijiu [6]. Besides, it has been shown that different enantiomers belonging to the same molecule may have different properties, including odor characteristics, odor thresholds, and even biological activities [7]. For example, *(R)*-3-mercapto-1-hexanol (odor threshold of 50 ng/L) had a pungent fruity aroma of grapefruit, while the *(S)*-configuration (odor threshold of 60 ng/L) was passion fruit notes [8,9].

As shown in Table 1, chiral compounds in alcoholic beverages usually used direct injection (DI), liquid-liquid extraction (LLE) and solid-phase microextraction (SPME), et al. pre-treatment, before being separated by chiral Gas Chromatography-Mass Spectrometry (GC-MS). Compared to SPME, DI has the advantage of being convenient and a direct reflection of the distribution of aroma components, and LLE has the advantage of enriching trace components [10]. After extraction of the chiral compounds, the volatile analytes are usually analyzed using GC-MS, which has the advantages of good reproducibility, high resolution and efficiency [11]. In addition to this, there are several methods such as solid-liquid extraction (SLE) and stir sorptive bar extraction (SBSE), and multidimensional gas chromatography coupled with a time-of-flight mass spectrometer (MDGC-TOF-MS) [12]. Different types of extraction and detection methods can be selected depending on the complexity of the sample and/or the concentration level of the analytes.

**Figure 1:**
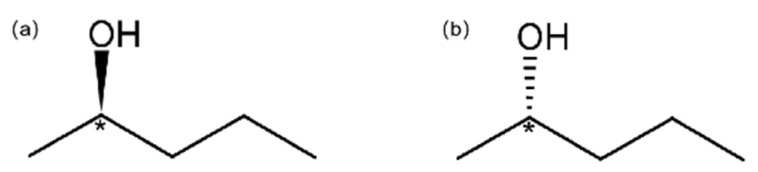
(**a**) *(R)*-2-Pentanol (CAS No. 31087-44-2), (**b**) *(S)*-2-Pentanol (CAS No. 26184-62-3).

Baijiu is a distilled spirit with complex flavors that are made from sorghum, wheat, rice, and other grains as the primary ingredients for brewing, with Daqu, Xiaoqu, and Fuqu serving as the saccharifying and fermenting agents, being extremely important to Chinese social culture [22,23]. Twelve different aroma types have been developed for Baijiu. Soy sauce aroma-type Baijiu (SSB), strong aroma-type Baijiu (STB), light aroma-type Baijiu (LTB), and rice aroma-type Baijiu (RTB) are the four basic aroma types, which are popular among consumers because of their unique flavors [24,25]. 2-Pentanol, also known as secondary amyl alcohol, is considered to be an important volatile substance in Moutai [26], Yanghe Daqu [27], Wuliangye [28], and red wine [29], etc.; it has an asymmetric chiral carbon atom and exists in two different stereoisomers, as shown in Figure 1, and its enantiomers are widely used in medicine, such as *(S)*-2-pentanol is a key chiral intermediate required for the synthesis of several potential anti-Alzheimer’s disease drugs [30]. Besides, novel chiral microemulsions using *(R)*-2-pentanol as surfactant are used in microemulsion electrokinetic chromatography for the enantiomeric separation of a variety of drugs [31]. The 2-pentanol enantiomer is the effective substrate in the key reaction of bacterial conversion of chiral epoxides to keto acids, with the Km of *(S)*-2-pentanol being 7.5 times higher than that of *(R)*-2-pentanol [32]; it was found that the *(R)*-2-pentanol conformation was predominantly present in purple passion fruit and as a near racemic mixture in yellow passion fruit [33] which might be suitable for distinguishing the two passion fruits. Given that the enantiomers of 2-pentanol may have different distribution and aroma characteristics, it is hypothesized that their different enantiomers may lead to different aroma profiles in Baijiu. Furthermore, to our knowledge, the distribution and content of 2-pentanol enantiomers in Baijiu and their sensory effects are unclear. Therefore, determining the enantiomeric distribution of 2-pentanol in different types of Baijiu is an important and valuable research topic. The objectives of this study were (i) to separate the stereoisomers of 2-pentanol in Baijiu by GC-MS using β-cyclodextrin as chiral stationary phases, (ii) to determine the enantiomeric distribution and content of 2-pentanol in SSB, STB, LTB and RTB, and (iii) to assess the odor characteristics and thresholds of 2-pentanol enantiomers by sensory analysis.

## 2. Materials and Methods

### 2.1. Chemicals

Anhydrous ethanol (chromatographic grade, 99.8%), Aladdin Reagent Co., Ltd. (Shanghai, China); *tert*-amyl alcohol (chromatographic grade, 98.0%), Macklin Technology Co., Ltd. (Shanghai, China); anhydrous sodium sulfate (99.0%) and sodium chloride (99.5%), Chengdu Jinshan Chemical Reagent Co., Ltd. (Chengdu, China); dichloromethane (99.9%), Shanghai Yien Chemical Technology Co., Ltd. (Shanghai, China); *(R)*-2-pentanol (chromatographic grade, 98.0%), *(S)*-2-pentanol (Chromatographic grade, 98.0%), Macklin Technology Co., Ltd. (Shanghai, China); distilled water, SZ-93A pure water distiller, Shanghai Yarong Co., Ltd. (Shanghai, China); TTL-DCI nitrogen blowing instrument, Beijing Tongtailian Technology Development Co., Ltd. (Beijing, China); nylon syringe filter, Guangzhou Pixillo Technology Co. (Guangzhou, China).

### 2.2. Sample

The *(R)*-2-pentanol and *(S)*-2-pentanol were determined in a total of 48 commercial Baijiu, including 19 types of SSB, 12 types of STB, 13 types of LTB, and 4 types of RTB. Samples information were shown in Table S1.

### 2.3. Stationary Phase Selection for Enantiomers of 2-Pentanol

Eight different chiral columns were selected, and the chiral column types were shown in Table 2. 2-Pentanol racemate was determined using a Thermo Fisher gas chromatograph (Trace 1300, Thermo Fisher, Waltham, Massachusetts, USA), and the best column was selected according to the separation resolution. Take 5 µL 2-pentanol racemate with anhydrous ethanol constant volume to 10 mL, filtered through a 0.22 µm nylon syringe filter membrane into the injection vial, and stored in a refrigerator at 4 °C until GC analysis. The 2-pentanol racemate was injected into the injection port in split mode (injector temperature 250 °C, split ratio: 20:1). The separation was carried out on eight different chiral columns under the same chromatographic conditions. GC conditions: column oven of 40 °C, held for 1 min, increased to 120 °C at a rate of 2 °C/min, and finally increased to 210 °C at 3 °C/min and held for 1 min. The carrier gas was nitrogen (99.999%) at a constant flow rate of 1 mL/min. Chromatograms were analyzed using Chromeleon 7 software (Thermo Fisher, Waltham, Massachusetts, USA).

### 2.4. Sample Pre-Treatment

#### 2.4.1. DI

3 mL sample was added directly to a 10 mL centrifuge tube, dried overnight with 1.5 g anhydrous sodium sulfate, and then 990 µL of the dried Baijiu sample and 10 µL of the *tert*-amyl alcohol (8.00 mg/L) internal standard solution were mixed and filtered through a 0.22 µm nylon syringe filter membrane into the injection vial and stored in a 4 °C refrigerator until GC-MS analysis.

#### 2.4.2. LLE

For the LLE the method used was based on a previously reported work [34], with some modifications. A 25 mL sample of the experimental Baijiu was diluted with water to 10% (*v/v*), with 500 µL of *tert*-amyl alcohol (1.00 mg/L) as the internal standard, and a certain amount of sodium chloride was weighed to saturate the solution, which was then transferred to a partition funnel and extracted with 10, 10, and 5 mL of dichloromethane. The organic phases were mixed after extraction, dried overnight with 15 g anhydrous sodium sulfate, and concentrated under nitrogen flow to obtain 0.5 mL of Baijiu extract, which was stored in a refrigerator at 4 °C until GC-MS analysis.

### 2.5. Separation and Quantification of 2-Pentanol Enantiomers in Baijiu by GC-MS

The enantiomers of 2-pentanol in Baijiu were determined by GC-MS (7890B GC-7000D MSD, Agilent, Santa Clara, California, USA). The pre-treated Baijiu sample was injected into the injection port in split mode (injector temperature 250 °C, split ratio: 20:1) The column was CYCLOSIL-B (30 m × 0.25 mm × 0.25 µm, Agilent Technologies Ltd., Santa Clara, CA, USA). The GC-MS column oven was started at 40 °C, increased to 50 °C at a rate of 0.5 °C/min, and finally increased to 210 °C at 6 °C/min and maintained for 1 min. The mass spectrometer was operated in the forward mode of the EI (Electron impact) ion source with electron impact ionization at 70 eV, ion source temperature 230 °C, quadrupole temperature 150 °C, emission current 0.35 μA and qualitative ions (m/z) of *(R)*- and *(S)*-2-pentanol were 75, 73, and 60 and quantitative ions (m/z) were 51. The carrier gas was helium (99.999%) at a constant flow rate of 1 mL/min. All experiments were repeated three times. The quantification was performed by using the internal standard curve method. Repeatability was assessed by the relative standard deviation of the concentrations of six independently tested Baijiu samples performed under the same analytical conditions. Precision was assessed by the relative standard deviation of the concentrations of the 2-pentanol enantiomeric standards in 6 times injections under the same analytical conditions. The detection limit was expressed as a concentration at three times the signal-to-noise ratio of the measurable peak. The recoveries were evaluated by adding the 2-pentanol enantiomers to Baijiu samples naturally containing low levels of this compound. Chromatograms were analyzed using the Agilent Masshunter workstation software (Agilent, Santa Clara, CA, USA).

### 2.6. Sensory Analysis

A panel of 10 trained judges (including 5 males and 5 females, aged 22–26 years) described the odor of 2-pentanol chiral standards.

According to GB/T33406-2016 [35], the olfactory threshold of 2-pentanol enantiomers were determined using a three-alternative forced-choice (3-AFC), where samples of different concentrations (approximately 10 mL) were prepared and poured into tulip Baijiu glasses and marked with random three-digit numbers. Assessments were performed in a dedicated room to prevent communication between assessors, under normal daylight and room temperature. The results of the olfactory threshold were analyzed according to the best estimate threshold (BET) method used.

### 2.7. Statistics and Analysis

Data were collated using Microsoft office excel 2018 (Microsoft, Redmond, Washington, USA), origin 2018 (64 Bit, OriginLab, Northampton, Massachusetts, USA) to produce box line plots, and the relationship between the concentrations of *(R)*- and *(S)*-2-pentanol in the Baijiu samples were evaluated using IBM SPSS Statistics25 (International Business Machines Corporation, Armonk, New York, USA) correlation test with a statistical significance level of 5%.

## 3. Results and Discussion

### 3.1. Stationary Phase Selection for 2-Pentanol Enantiomers

**Figure 2:**
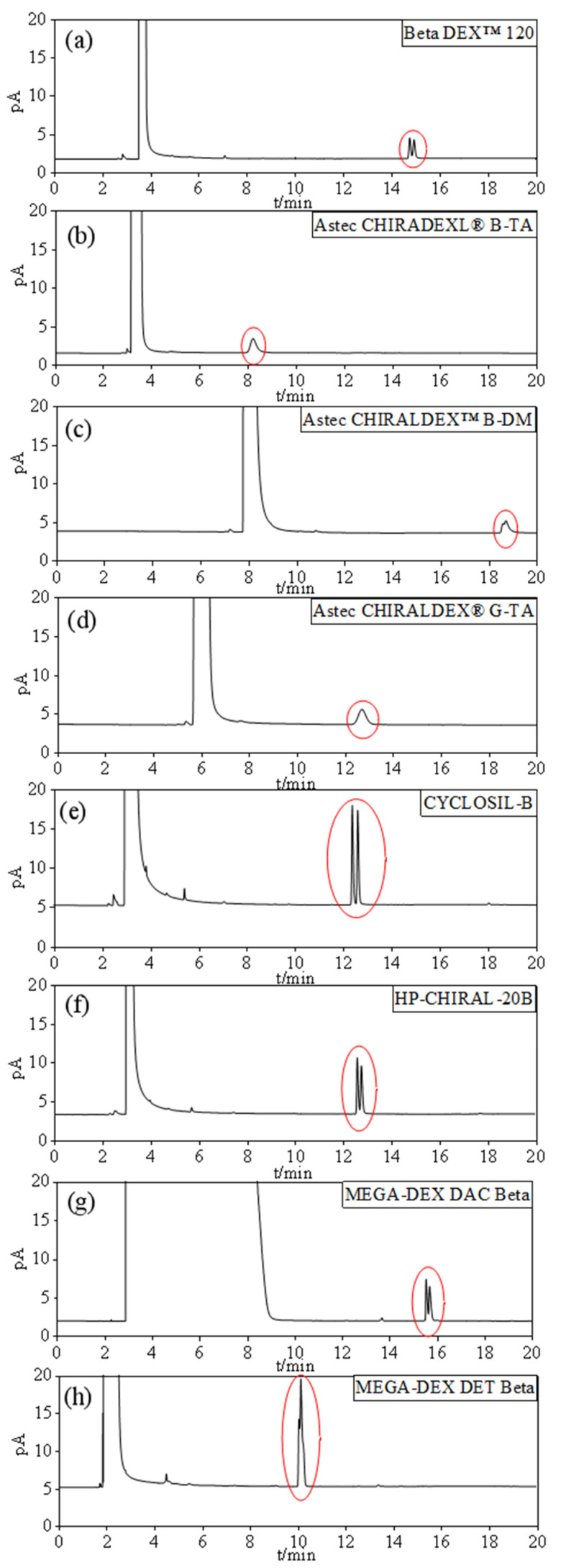
Chromatograms of 2-pentanol racemate separated by different chiral chromatographic columns (**a**): Beta DEX™ 120, (**b**): Astec CHIRADEXL^®^ B-TA, (**c**): Astec CHIRALDEX™ B-DM, (**d**): Astec CHIRALDEX^®^ G-TA, (**e**): CYCLOSIL-B, (**f**): HP-CHIRAL-20B, (**g**): MEGA-DEX DAC Beta, (**h**): MEGA-DEX DET Beta.

Figure 2 illustrated the separation of 2-pentanol racemate on different columns (different stationary phases) under the same chromatographic conditions. The best separation resolution (Rs, 1.92) and intensities were achieved on the CYCLOSIL-B capillary column (Figure 2e). Therefore, this column was chosen for the separation and quantification of 2-pentanol enantiomeric in Baijiu.

According to our investigation, the separation of 2-butanol and 2,3-butanediol isomers in wine [20], and 1,2-propanediol enantiomers in Baijiu [6] have been successfully separated using hepta-(2,3-di-O-methyl-6-O-*tert*-butyldimethylsilyl)-β-cyclodextrin (CYCLOSIL-B column) as stationary phase; it is assumed that this stationary phase has a good result on chiral alcohol separation.

### 3.2. Effect of DI and LLE on the Enantiomeric Detection of 2-Pentanol in Baijiu

Enantiomeric analysis of 2-pentanol in Baijiu using DI and LLE were conducted. The standard curves for 2-pentanol were established by DI with the correlation coefficients of 0.9997 and 0.9991 for *(R)*- and *(S)*-2-pentanol, respectively, and the limits of detection (LOD) of 0.65 mg/L and 0.35 mg/L, with recoveries ranging from 90.69~105.28% and 92.02~98.97%, respectively. The standard curves for 2-pentanol were established by LLE, and the coefficients of determination were 0.9998 for both *(R)*- and *(S)*-2-pentanol, and the limits of detection were 0.03 mg/L and 0.02 mg/L for *(R)*- and *(S)*-2-pentanol, respectively, with the recoveries ranging from 76.76~100.03% and 74.60~121.47%. The relative standard deviations of the two enantiomers for the reproducibility and precision of the two pre-treatments were below 10%, as shown in Table 3. The results showed that both pre-treatment methods showed good satisfactory accuracy, precision and sensitivity for the determination of the enantiomeric separation of 2-pentanol in Baijiu.

**Figure 3:**
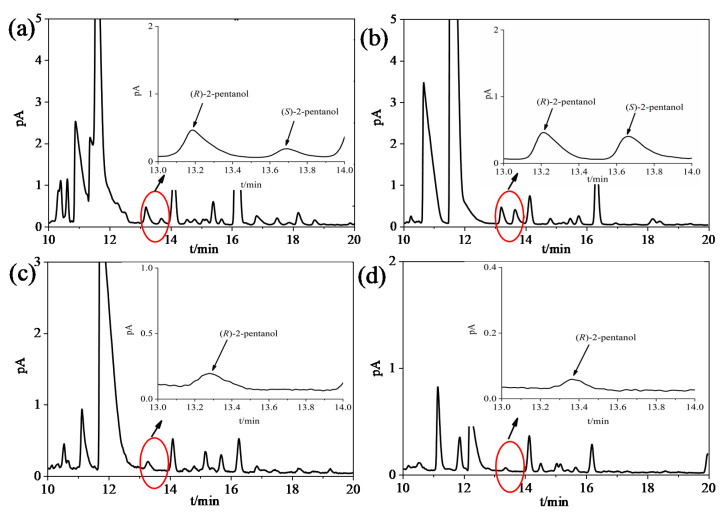
Enantiomeric separation chromatograms of 2-pentanol in representative Baijiu samples of different aroma types using LLE (**a**): SSB, (**b**): STB, (**c**): LTB, (**d**): RTB.

Two pre-treatments were used to analyze the 2-pentanol enantiomers in Baijiu. Figure 3 and Figure S1 showed the chromatograms of four representative Baijiu samples by LLE and DI, respectively, which exhibited good separation of the 2-pentanol enantiomers in Baijiu.

**Figure 4:**
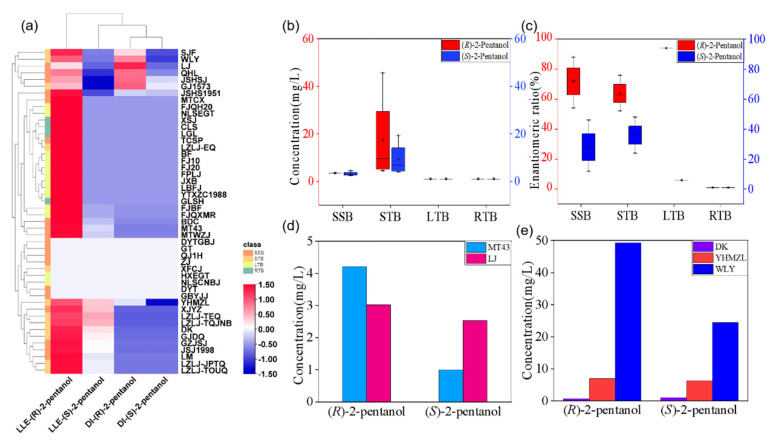
(**a**): 2-pentanol enantiomers concentration heat map, (**b**): Double y-axis box line plot (Red coordinates were *(R)*-2-pentanol, blue coordinates were *(S)*-2-pentanol) of 2-pentanol isomer concentration using LLE, (**c**): Double y-axis box line plot of 2-pentanol enantiomeric ratio using LLE, (**d**) Box Line plot of 2-pentanol enantiomer content in MT43 and LJ, (**e**) Box Line plot of 2-pentanol enantiomer content in DK, YHMZL, and WLY.

LLE combined with GC-MS was used for quantitative analysis of 2-pentanol enantiomers in Baijiu, and the detection results were shown in Table 4. The detailed DI section data were shown in Table S2. The results demonstrated that the *(R)*-2-pentanol was preponderant in Baijiu. 2-Pentanol was detected in 39 types of commercial Baijiu by LLE (Table 5). The mean concentration of *(R)*-2-pentanol by LLE were 3.24 (±0.30) mg/L, 7.74 (±1.40) mg/L, 0.68 (±0.07) mg/L and 1.09 (±0.15) mg/L in SSB, STB, LTB and RTB, respectively. Only *(R)*-configuration was found in RTB. The mean concentrations of *(S)*-2-pentanol were 1.41 (±0.13) mg/L, 4.76 (±0.85) mg/L, 0.02 (±0.01) mg/L in SSB, STB, and LTB, respectively. The average enantiomeric ratios *R:S* were 72:28, 64:36, and 94:6 in SSB, STB, and LTB, respectively. 2-Pentanol was detected by DI in only 8 types of 48 commercial Baijiu and was not detected in either LTB or RTB. Compared to DI, LLE is more suitable for the enantiomeric determination of 2-pentanol in Baijiu (Figure 4a).

### 3.3. Separation and Quantification of 2-Pentanol Enantiomers in SSB, STB, LTB, and RTB

The enantiomers of 2-pentanol were separated by GC-MS during the β-cyclodextrin separation phase and their distribution and concentration in SSB, STB, LTB, and RTB were investigated. The analysis of 48 commercial Baijiu of four aromatic types showed that their distribution and concentration were different.

The highest content of *(R)*-2-pentanol in STB was 0.62 (±0.03)~49.30 (±10.54) mg/L, followed by SSB and RTB with 0.68 (±0.08)~6.18 (±0.25) mg/L and 0.98 (±0.06)~1.34 (±0.41) mg/L, respectively, while the content in LTB was very low at 0.41 (±0.00)~0.85 (±0.05) mg/L. The highest level of *(S)*-2-pentanol in STB was 0.33 (±0.07)~24.43 (±3.97) mg/L, followed by SSB was 0.29 (±0.03)~3.38 (±0.95) mg/L, while the very low level in LTB was 0.03 (±0.01)~0.04 (±0.01) mg/L. In RTB *(S)*-enantiomer was not detected. Based on the quantitative results, *(R)*- and *(S)*-2-pentanol concentrations were significantly higher in STB than in SSB, LTB, and RTB (Figure 4b,c).

SSB is made from sorghum, which is fermented eight times and distilled seven times by high-temperature yeast and has an elegant aroma, full-bodied sweetness, and a long-lasting aftertaste [36]. The concentration and enantiomeric ratios of *(R)*- and *(S)*-2-pentanol in SSB were different, and the *(R)*-configuration dominated. In this study, the *R:S* enantiomeric ratio of MT43 (Maotai) from Guizhou province was 81:19 and that of LJ (Langjiu) from Sichuan province was 54:46 (Figure 4d), with large differences in the enantiomeric ratio of 2-pentanol in the same aromatic Baijiu from different regions, which might be caused by geographical differences.

STB is the most produced type of Baijiu in China, which is typically characterized by strong cellar aroma, purity, harmonious aroma, and long aftertaste [37]. STB can be divided into single-grain STB and multigrain STB according to raw materials. Single-grain STB is made from sorghum as a single raw material, such as LZLJ. The multigrain STB is made from five grains (wheat, rice, corn, sorghum, and glutinous rice), such as WLY [10]. There was a significant difference (*p* < 0.05) in the ratio of 2-pentanol enantiomers comparing WLY and LZLJ, which led to the assumption that the difference in 2-pentanol enantiomers was related to the raw materials used in the production of Baijiu. According to the aroma characteristics of different geographical locations, STB can be divided into three major categories, namely Sichuan, Jianghuai, and Northern [38], comparing representative STB brands from three representative regions (i.e., WLY, YHMZL, and DK), it was found that the enantiomeric concentrations of 2-pentanol in them were different (Figure 4e) (*p* < 0.05), and the reason for this might be related to the fermentation temperature in different regions.

LTB is produced using sorghum as raw material, barley, and pea powder for the production of the crank, and has an elegant and harmonious ethyl acetate aroma; it is widely popular among consumers for its pleasant fruity and floral aromas [34,39,40]. *(R)*-2-Pentanol was detected in 11 LTB Baijiu samples, and *(S)*-enantiomer only in 2 Baijiu samples by LLE, the average ratio of *R:S* was 94:6. Among the four major aromatic types of Baijiu, RTB is the only one that utilizes rice as a raw material and liquid fermentation by saccharification with Xiaoqu [41]. Only *(R)*-2-pentanol was detected in the studied RTB. The particular enantiomeric distribution of *(R)*-2-pentanol with the great predominance of *(R)*-enantiomer in LTB and RTB could be a potential marker for the adulteration.

The results showed that the enantiomers of 2-pentanol were discrepant in different aroma types of Baijiu. The concentration and distribution differences of 2-pentanol enantiomers in Baijiu showed that their sources were complex. Firstly, there were differences in microbial communities and enzymes of Baijiu fermentation [42]. Secondly, Baijiu fermentation is a unique and complex process. Saccharification and spontaneous fermentation are carried out at the same time, and different fermentation temperatures cause the growth and reproduction of different microorganisms [43]. Finally, Baijiu aging will also have different effects under different environmental conditions. Some studies have shown that alcohols in Baijiu originate from glucose anabolism or amino acid catabolism, or are produced by yeast using the Ehrlich pathway [44]; however, the origin of 2-pentanol enantiomers is unknown, and the elucidation of its specific metabolic pathway requires specific studies.

### 3.4. Sensory Analysis of Enantiomers of 2-Pentanol

The 10 judges described that *(R)*-2-pentanol presented a note of paint, rubber, and grease, while *(S)*-configuration was characterized by mint, plastic, and pungent notes (Table 6); this differed from the odor description of the aroma by Brenna et al. [45]; however, 10 judges and Brenna et al. all agreed that *(S)*-2-pentanol showed a stronger aroma.

The olfactory thresholds were established in pure water and 46% ethanol solutions using 3-AFC. The olfactory threshold of *(R)*-2-pentanol was 12.62 mg/L, which was about 4 times that of *(S)*-enantiomer (3.03 mg/L) in pure water. The olfactory threshold of *(R)*-2-pentanol was 163.30 mg/L and *(S)*-enantiomer was 78.58 mg/L in 46% ethanol solution, and the olfactory threshold of *(R)* in 46% ethanol was about 2 times that of *(S)*. The olfactory threshold of 2-pentanol racemate in 46% ethanol aqueous solution was reported to be 194 mg/L [46], and the olfactory thresholds of both *(R)*- and *(S)*-enantiomer were lower than the racemate. The olfactory thresholds of *(S)*-configuration were lower than that of *(R)*-configuration in both mediums, suggesting that their thresholds might be dependent on stereochemical structure. Sensory evaluation of individual stereoisomers of 2-pentanol extended the knowledge of the effect of their configuration on sensory perception.

## 4. Conclusions

The study on the content distribution of 2-pentanol enantiomers in four aroma types of Baijiu complemented the existing knowledge of 2-pentanol in Baijiu. The results showed that *(R)*-2-pentanol was predominant in Baijiu and the *R:S* ratios were different. 2-Pentanol was detected in 39 Baijiu by LLE, the average enantiomeric ratios *R:S* were 72:28, 64:36, and 94:6 in SSB, STB, and LTB, respectively; only the *(R)*-conformation was detected in RTB. As most Baijiu did not contain high levels of 2-pentanol and therefore LLE was more suitable for the enantiomeric analysis of 2-pentanol in Baijiu. By sensory analysis, 2-pentanol enantiomers had different odor characteristics, with different olfactory thresholds in pure water and 46% ethanol solutions. *(R)*-2-pentanol was described as paint, rubber, and grease, while the *(S)*-type had mint, plastic, and pungent notes. The olfactory thresholds of *(R)*-form and *(S)*-form were 163.30 mg/L and 78.58 mg/L in 46% ethanol and 12.62 mg/L and 3.03 mg/L in pure water, respectively. In the future, based on a large amount of systematic data, the difference in the enantiomeric ratio of 2-pentanol may be used in Baijiu as a basis for tracing its adulteration.

## 5. Patents

There are patents resulting from the work reported in this manuscript.

## Figures and Tables

**Table 1:** The respective methodologies of analysis for chiral compounds of alcoholic beverages.

Methodologies	Samples	Chiral Compounds
DI-GC, DI-GC-FID	Wine [4], Baijiu [6]	ethyl lactate, 1,2-propanediol
LLE-GC-MS, LLE-MDGC-TOF-MS	Wine [13,14], white wine [8,15], red wine [16,17], Bordeaux dessert wines [18]	3-mercapto-1-hexanol, 3-mercaptohexyl Acetate, 2-methylbutyl acetate, 2-methylbutyric acid, 3-hydroxybutyric acid, 2-hydroxy-3-methylbutyric acid, 2-hydroxy-4-methylvaleric acid, 2-nonen-4-olide, *γ*-nonalactone, 3-methyl-4-octanolide, ethyl 3-Hydroxybutanoate, ethyl 2-hydroxy-3-methylbutanoate, (3-hydroxy-4, 5-dimethyl-2 (5H)-furanone)
SPME-GC-MS, SPME-GC	fruit brandy [19], wine [20,21]	linalool, *trans*-linalool oxide, *cis*-linalool oxide, limonene, *α*-terpineol, nerolidol, *β*-citronellol, *γ*-decalactone, *γ*-dodecalactone, 2-Butanol, 2,3-Butanediol, *α*-ionone

**Table 2:** Different column types.

Chromatographic Column Names	Stationary Phase	Specification
Beta DEX™ 120	SPB-35 poly (35% phenyl/65% methylsiloxane) containing 20% permethylated β-cyclodextrin	30 m × 0.25 mm × 0.25 µm
Astec CHIRADEXL^®^ B-TA	2,6-di-O-pentyl-3-trifluoroacetyl derivative of β-cyclodextrin	30 m × 0.25 mm × 0.25 µm
Astec CHIRALDEX™ B-DM	2,3-di-O-methyl-6-t-butyl silyl derivative of β-cyclodextrin	50 m × 0.25 mm × 0.12 µm
Astec CHIRALDEX^®^ G-TA	2,6-di-O-pentyl-3-trifluoroacetyl derivative of γ-cyclodextrin	30 m × 0.25 mm × 0.25 µm
CYCLOSIL-B	hepta-(2,3-di-O-methyl-6-O-*tert*-butyldimethylsilyl)-β-cyclodextrin	30 m × 0.25 mm × 0.25 µm
HP-CHIRAL-20B	(35%-phenyl)-methyl polysiloxane-β-cyclodextrin	30 m × 0.25 mm × 0.25 µm
MEGA-DEX DAC Beta	diacetyl tertbutylsilyl-β-cyclodextrin	30 m × 0.25 mm × 0.25 µm
MEGA-DEX DET Beta	diethyl-TBS-β-cyclodextrin	30 m × 0.25 mm × 0.25 µm

**Table 3:** Linear range, RSD, LOD, and recovery rate of 2-pentanol in GC-MS analysis.

Pre-Process	Compounds	Linearity Range (mg/L)	R^2^	RSD (%)	LODs(mg/L)	Recovery Rate (%)
DI	*(R)*-2-Pentanol	2.60~253.70	0.9997	0.71~9.75%	0.65	90.69~105.28%
	*(S)*-2-Pentanol	1.30~126.85	0.9991	0.00~8.75%	0.35	92.02~98.97%
LLE	*(R)*-2-Pentanol	0.08~79.28	0.9998	2.56~6.64%	0.03	76.76~100.03%
	*(S)*-2-Pentanol	0.03~29.73	0.9998	2.62~5.19%	0.02	74.60~121.47%

Note: R^2^: correlation coefficient, LOD: limit of detection, RSD: relative standard deviation.

**Table 4:** Enantiomeric content and ratio of 2-pentanol in Baijiu analyzed by LLE-GC-MS.

Samples	*(R)*-2-Pentanol (mg/L)	*(S)*-2-Pentanol (mg/L)	ee	*R:S*
SSB				
BDC	2.06 ± 0.77 a	0.29 ± 0.03 a	75.32%	88:12
DYT	-	-	-	-
GZJSJ	3.85 ± 1.00 ab	1.40 ± 0.33 a	46.67%	73:27
GBYJJ	-	-	-	-
DYTGBJ	-	-	-	-
GT	-	-	-	-
JSHS1951	6.10 ± 0.43 ab	2.28 ± 0.19 a	45.58%	73:27
JSHSJ	3.06 ± 0.10 ab	1.40 ± 0.02 a	37.22%	69:31
JSJ1998	2.56 ± 0.27 ab	1.00 ± 0.11 a	43.82%	72:28
LM	1.92 ± 0.00 a	0.54 ± 0.05 a	56.10%	78:22
LJ	3.02 ± 0.06 ab	2.53 ± 0.32 a	8.83%	54:46
MT43	4.22 ± 0.31 ab	1.00 ± 0.04 a	61.69%	81:19
MTCX	0.68 ± 0.08 a	-	-	-
MTWZJ	6.18 ± 0.25 ab	1.23 ± 0.08 a	66.80%	83:17
QJ1H	-	-	-	-
QHL	3.51 ± 0.01 ab	2.93 ± 0.07 a	9.01%	55:45
TCSP	3.41 ± 0.35 ab	-	-	-
XJYZ	1.52 ± 0.28 a	0.91 ± 0.19 a	25.10%	63:37
ZJ	-	-	-	-
STB				
DK	0.74 ± 0.06 a	0.36 ± 0.06 a	34.55%	67:33
GJDQ	0.89 ± 0.06 a	0.38 ± 0.07 a	40.16%	70:30
GJ1573	4.15 ± 0.05 ab	3.39 ± 0.46 ab	10.08%	55:45
LZLJ-JPTQ	1.20 ± 0.05 a	0.38 ± 0.05 a	51.90%	76:24
LZLJ-EQ	0.62 ± 0.03 a	-	-	-
LZLJ-TEQ	1.74 ± 0.14 a	1.24 ± 0.09 a	16.78%	58:42
LZLJ-TQJNB	1.55 ± 0.12 a	1.04 ± 0.07 a	19.69%	60:40
LZLJ-TOUQ	1.04 ± 0.13 a	0.33 ± 0.07 a	51.82%	76:24
SJF	16.94 ± 2.58 c	9.74 ± 1.87 d	26.99%	63:37
WLY	49.30 ± 10.54 d	24.43 ± 3.97 e	33.73%	67:33
XFCJ	-	-	-	-
YHMZL	6.96 ± 1.60 ab	6.34 ± 1.82 bc	4.66%	52:48
LTB				
BF	0.85 ± 0.05 a	-	-	-
FJ10	0.69 ± 0.03 a	-	-	-
FJ20	0.65 ± 0.04 a	-	-	-
FJBF	0.48 ± 0.03 a	0.03 ± 0.01 a	88.24%	94:6
FJQH20	0.78 ± 0.08 a	-	-	-
FJQXMR	0.62 ± 0.04 a	0.04 ± 0.01 a	87.88%	94:6
FPLJ	0.44 ± 0.06 a	-		
HXEGT	-	-	-	-
JXB	0.84 ± 0.19 a	-		
LBFJ	0.66 ± 0.02 a	-	-	-
YTXZC1988	0.41 ± 0.00 a	-	-	-
NLSEGT	0.78 ± 0.07 a	-	-	-
NLSCNBJ	-	-	-	-
RTB GLSH				
	1.04 ± 0.08 a	-	-	-
XSJ	0.98 ± 0.06 a	-	-	-
CLS	1.00 ± 0.05 a	-	-	-
LGL	1.34 ± 0.41 a	-	-	-

Note:”-” means not detected; the significant difference between data with different letters in the same column (*p* < 0.05).

**Table 5:** Enantiomeric concentrations and distribution of 2-pentanol in four types of Baijiu with LLE.

		Mean Concentration (mg/L) ± Standard Deviation			
Aroma-Types	Number	*RS*	*R*	*S*	*R:S*
SSB	13	4.65 ± 0.46	3.24 ± 0.30	1.41 ± 0.13	72:28
STB	11	12.50 ± 1.13	7.74 ± 1.40	4.76 ± 0.85	64:36
LTB	11	0.70 ± 0.04	0.68 ± 0.07	0.02 ± 0.01	94:6
RTB	4	1.09 ± 0.15	1.09 ± 0.15	-	100:0

**Table 6:** Odor description and olfactory thresholds of 2-pentanol enantiomers.

			Olfactory Threshold (mg/L)	
Compound	Odor Description	Odor Description [45]	In Pure Water	In 46% Ethanol Solution
*(R)*-2-Pentanol	Paint, rubber, grease	Light, seedy, sharp	12.62	163.30
*(S)*-2-Pentanol	Mint, plastic, pungent	Heavy, wild berry, ripe, dusty, astringent	3.03	78.58

## Data Availability

The data presented in this study are available on request from the corresponding author. The data are not publicly available due to patents which are resulting from this study.

## Supplementary Materials

The following supporting information can be downloaded at: https://www.mdpi.com/article/10.3390/foods11172584/s1, Table S1: Baijiu samples information, Figure S1: Enantiomeric separation chromatograms of 2-pentanol in representative Baijiu samples of different aroma types using DI (a): SSB, (b): STB, (c): LTB, (d): RTB. Table S2: Enantiomeric contents and ratios of 2-pentanol in Baijiu analyzed by DI-GC-MS.
