# Supplementary material for: Determination of 2-Pentanol Enantiomers via Chiral GC-MS and Its Sensory Evaluation in Baijiu
Source: Foods. 2022 Aug 26;11(17):2584. doi: 10.3390/foods11172584 (PMC9455680; doi:10.3390/foods11172584)
Supplement: Supplementary file 1 [file foods-11-02584-s001.zip › foods-1863826-supplementary.pdf]

Supplementary Materials

Determination of 2-pentanol enantiomers via chiral GC-MS and its sensory evaluation in Baijiu

Lisha Hu, Shuyi Qiu, Yifeng Dai *, Luqin Tian, Chaoyang Wei

* Correspondence: yfdai3@gzu.edu.cn

Contents

Table S1 Baijiu samples information.. 3

Figure S1. Enantiomeric separation chromatograms of 2-pentanol in representative Baijiu samples of different aroma types using DI (a): SSB, (b): STB, (c): LTB, (d): RTB 5

Table S2 Enantiomeric contents and ratios of 2-pentanol in Baijiu analyzed by DI-GC-MS..... 6

Table S1 Baijiu samples information

Samples	origin of production	year of production
**SSB**		
BDC	Heilongjiang Province, China	2021
DYT	Guizhou province, China	2020
GZJSJ	Guizhou province, China	2018
GBYJJ	Guizhou province, China	2016
DYTGBJ	Guizhou province, China	2018
GT	Guizhou province, China	2018
JSHS1951	Guizhou province, China	2020
JSHSJ	Guizhou province, China	2018
JSJ1998	Guizhou province, China	2019
LM	Heilongjiang province	2020
LJ	Sichun province, China	2018
MT43	Guizhou province, China	2020
MTCX	Guizhou province, China	2017
MTWZJ	Guizhou province, China	2018
QJ1H	Guizhou province, China	2017
QHL	Sichun province, China	2020
TCSP	Guizhou province, China	2017
XJYZ	Guizhou province, China	2020
ZJ	Guizhou province, China	2018
**STB**		
DK	Henan province, China	2020
GJDQ	Anhui province, China	2021
GJ1573	Sichun province, China	2020
LZLJ-JPTQ	Sichun province, China	2020
LZLJ-EQ	Sichun province, China	2017
LZLJ-TEQ	Sichun province, China	2018
LZLJ-TQJNB	Sichun province, China	2020
LZLJ-TOUQ	Sichun province, China	2017
SJF	Sichun province, China	2021
WLY	Sichun province, China	2020
XFCJ	Sichun province, China	2020
YHMZL	Jiangsu province, China	2021
**LTB**		
BF	Henan province, China	2020
FJ10	Shanxi province, China	2021
FJ20	Shanxi province, China	2021
FJBF	Shanxi province, China	2020
FJQH20	Shanxi province, China	2020
FJQXMR	Shanxi province, China	2020
FPLJ	Shanxi province, China	2019
HXEGT	Beijing, China	2019
JXB	Chongqing, China	2019

Samples	origin of production	year of production
LBFJ	Shanxi province, China	2020
YTXZC1988	Shanxi province, China	2018
NLSEGT	Beijing, China	2019
NLSCNBJ	Beijing, China	2019
**RTB**		
GLSH	Guangxi province, China	2021
XSJ	Guangxi province, China	2021
CLS	Guangdong province, China	2021
LGL	Guangxi province, China	2021

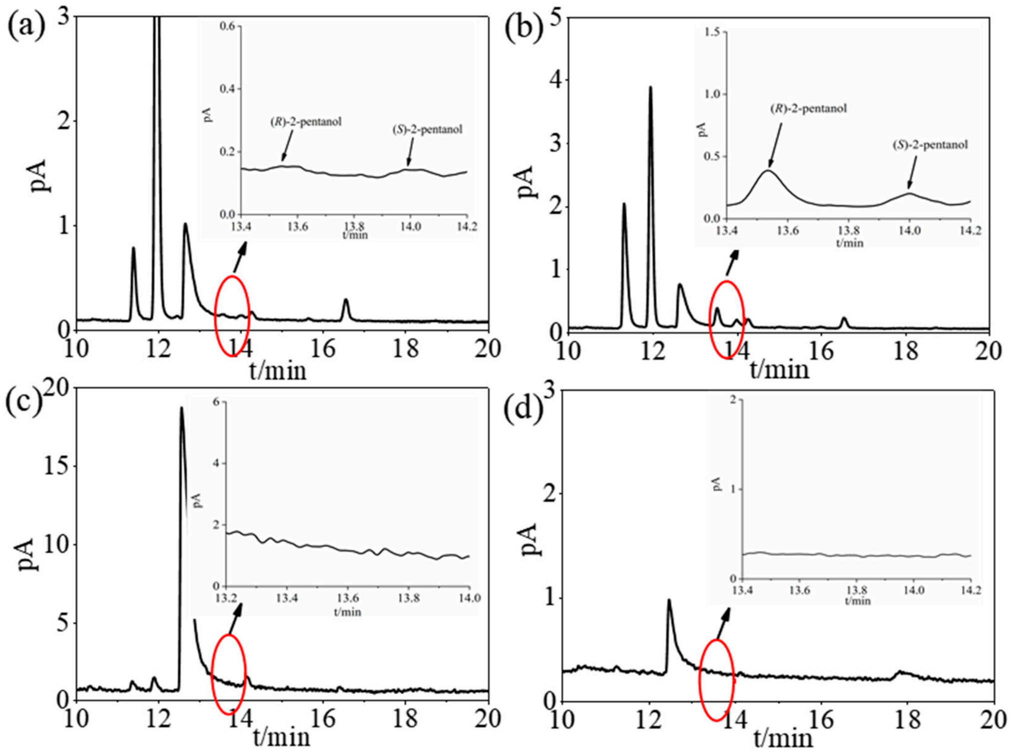

Figure S1. Enantiomeric separation chromatograms of 2-pentanol in representative Baijiu samples of different aroma types using DI (a): SSB, (b): STB, (c): LTB, (d): RTB

Table S2 Enantiomeric contents and ratios of 2-pentanol in Baijiu analyzed by DI-GC-MS

Samples	*(R)*-2-pentanol (mg/L)	*(S)*-2-pentanol (mg/L)	ee	*R:S*
**SSB**				
BDC	-	-	-	-
DYT	-	-	-	-
GZJSJ	-	-	-	-
GBYJJ	-	-	-	-
DYTGBJ	-	-	-	-
GT	-	-	-	-
JSHS1951	3.50±0.23a	3.38±0.95b	1.74%	51:49
JSHSJ	3.34±0.20a	2.42±0.29a	15.97%	58:42
JSJ1998	-	-	-	-
LM	-	-	-	-
LJ	3.51±0.20a	2.55±0.12a	15.84%	58:42
MT43	-	-	-	-
MTCX	-	-	-	-
MTWZJ	-	-	-	-
QJ1H	-	-	-	-
QHL	3.53±0.38a	3.08±0.30ab	6.81%	53:47
TCSP	-	-	-	-
XJYZ	-	-	-	-
ZJ	-	-	-	-
**STB**				
DK	-	-	-	-
GJDQ	-	-	-	-
GJ1573	4.37±0.12a	3.94±0.38ab	5.17%	53:47
LZLJ-JPTQ	-	-	-	-
LZLJ-EQ	-	-	-	-
LZLJ-TEQ	-	-	-	-
LZLJ-TQJNB	-	-	-	-
LZLJ-TOUQ	-	-	-	-
SJF	13.24±0.52a	8.74±0.36c	20.47%	60:40
WLY	45.66±6.03b	19.35±1.94d	40.47%	70:30
XFCJ	-	-	-	-
YHMZL	5.91±0.28a	4.73±0.43b	11.09%	56:44
**LTB**				
BF	-	-	-	-
FJ10	-	-	-	-
FJ20	-	-	-	-
FJBF	-	-	-	-
FJQH20	-	-	-	-
FJQXMR	-	-	-	-

**Samples**	***(R)*-2-pentanol (mg/L)**	***(S)*-2-pentanol (mg/L)**	**ee**	***R:S***
FPLJ	-	-	-	-
HXEGT	-	-	-	-
JXB	-	-	-	-
LBFJ	-	-	-	-
YTXZC1988	-	-	-	-
NLSEGT	-	-	-	-
NLSCNBJ	-	-	-	-
**RTB**				
GLSH	-	-	-	-
XSJ	-	-	-	-
CLS	-	-	-	-
LGL	-	-	-	-

Note:"-" means not detected; the significant difference between data with different letters in the same column ($p < 0.05$).
